# The Effect of Direct and Indirect Monitoring on Generosity Among Preschoolers

**DOI:** 10.1038/srep09025

**Published:** 2015-03-12

**Authors:** Takayuki Fujii, Haruto Takagishi, Michiko Koizumi, Hiroyuki Okada

**Affiliations:** 1 Graduate School of Brain Science, Tamagawa University; 2 Tamagawa University Brain Science Institute; 3 Research Center for Child Mental Development, University of Fukui

## Abstract

The purpose of this study is to examine the effect of direct and indirect monitoring on generosity among five-year-old preschoolers and to reveal the primary motivation for their generosity. Forty-two preschoolers completed one-shot dictator games in Condition 1 while being monitored by the experimenter (the direct monitoring condition). In Condition 2, an image of staring eyes was displayed on the computer monitor (the indirect monitoring condition). In Condition 3, the computer monitor showed a picture of flowers (the non-monitoring condition). The results showed that while there was no difference between the mean levels of allocation in the indirect and non-monitoring conditions, the mean level of allocation in the direct monitoring condition was significantly higher than in the non-monitoring condition. These results showed that five-year-old preschoolers concerned with being monitored by, and receiving direct responses from, others tend to be more generous.

## Introduction

Concern for personal reputation is a strong behavioural incentive in both industrial and traditional societies alike^1^; thus, being monitored by others leads us to behave more generously^2^,^3^. Izuma, et al.^2^ showed people tended to donate more money to charitable organizations when being monitored by others. That is, when other people are watching us, we are more strongly motivated to care about our own reputations. Previous studies on generosity have examined the differences in the effects of actual human monitoring^2^,^3^ compared to viewing screen images of either eyes or three dots^4^,^5^,^6^,^7^,^8^,^9^. In this paper, we term the latter ‘indirect monitoring’ and refer to the former as ‘direct monitoring’.

Does young children's generosity exhibit the effects of direct or indirect monitoring? Some developmental studies have examined the effect of direct monitoring on generosity^10^,^11^,^12^,^13^. Houser, et al.^12^ administered an economic social dilemma game to six- to eleven-year-old children and found that their contributions to other group members increased significantly when other people were watching. This effect was observed only for children aged nine years and older, suggesting that these children contributed to the group in order to either acquire a good reputation or to avoid a bad reputation.

It is reasonable to assume that older children behave in a pro-social manner in order to maintain a positive (or avoid a negative) reputation in the eyes of others because higher-order theory of mind (ToM; e.g., second-order false belief understanding) plays an important role in one's self-representation of their reputation^14^,^15^. After developing higher-order ToM, children care more about social evaluations^16^. Research examining pass rate in a second-order false belief task, which measures higher-order ToM, has shown that second-order false belief understanding becomes possible at around nine years of age^17^. Although one study demonstrated that even preschoolers can pass a simplified version of the second-order false belief task^18^, other studies have reported that nearly all four- and five-year-old children fail it^19^.

Recently, three studies^10^,^11^,^13^ reported that even five-year-old preschoolers shared more of their resources with others when a peer observer was present, and the tendency to give to others was significantly higher when in-group members, as opposed to out-group members, were the observers. However, these studies did not examine the relationship between the development of higher-order ToM and preschoolers' resource allocation. Thus, other motivation besides reputation concern may play an important role in five-year-old children's displays of generosity.

What is the primary motivation for preschoolers to show generosity? The previously mentioned developmental study showed that five-year-old children performed fair allocation during the ultimatum game, a simple economic game, in order to avoid being punished by their opponents^20^,^21^. In this study, children played the ultimatum game in a face-to-face setting; thus, the children likely cared about the reactions of the opponent in front of them. The results of these studies indicate that five-year-old children react to the direct responses of the people with whom they are interacting. Indeed, children tend to avoid being scolded by others and seek to receive praise in their daily lives. In this way, direct response concern, but not reputation concern, plays an important role in generosity among five-year-old children. Therefore, we hypothesize that the primary motivation for a five-year-old's generosity is not reputation concern, but concern for the direct response from others. To test this hypothesis, we conducted a dictator game (DG) with five-year-old children in the following three conditions:

  1) Direct monitoring condition: An experimenter watches the children.

  2) Indirect monitoring condition: An image of eyes is displayed on a computer monitor in an anonymous experimental situation.

  3) Non-monitoring condition: A picture of flowers is displayed on a computer monitor in an anonymous experimental situation.

Furthermore, we examined the development of children's first- and second-order false beliefs to confirm whether they are able to care about their own reputations.

### Prediction 1 (The effect of indirect monitoring on generosity)

First, we examined the effect of indirect monitoring (eye spots effect) on generosity among children. Previous studies showed that the presentation of an image of eyes enhances a person's motivation for building a good reputation^7^. If five-year-old children care about their own reputations, the mean level of allocation in the indirect monitoring condition will be significantly higher than in the non-monitoring condition. However, we predicted no differences between the two conditions, because almost no five-year-old child is able to care about his/her reputation, in the sense that he/she likely has not gained understanding of second-order false beliefs.

### Prediction 2 (The effect of direct monitoring on generosity)

Second, we examined the effect of direct monitoring on children's generosity. Previous studies^10^,^11^,^13^ have shown that five-year-old children become more generous when they are being watched. If children care about the direct response they receive from others, the mean level of allocation displayed by the children in the direct monitoring condition should be significantly higher than that displayed by the children in the non-monitoring condition. Previous studies have already examined Prediction 2^10^,^11^,^13^, so we compared the mean level of allocation between the two conditions to reconfirm the effect of direct monitoring on generosity in five-year-old children.

### Prediction 3 (Second-order false belief task)

Third, we examined the pass rate of the second-order false belief task among the five-year-old children. The ability to care about one's reputation requires an understanding of second-order false beliefs; therefore, we predicted that five-year-old children would fail this task.

## Results

**Figure 1:**
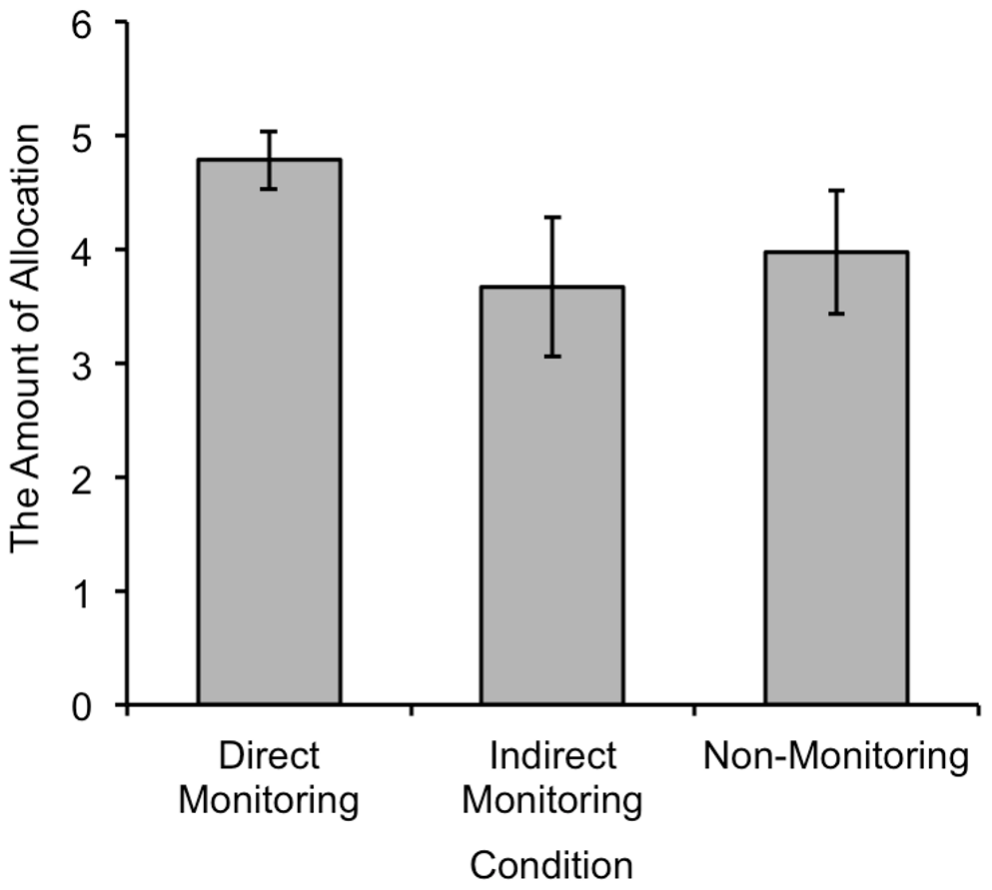
Mean level of allocation in each condition. The error bar indicates the 95% confidence interval.

**Figure 2:**
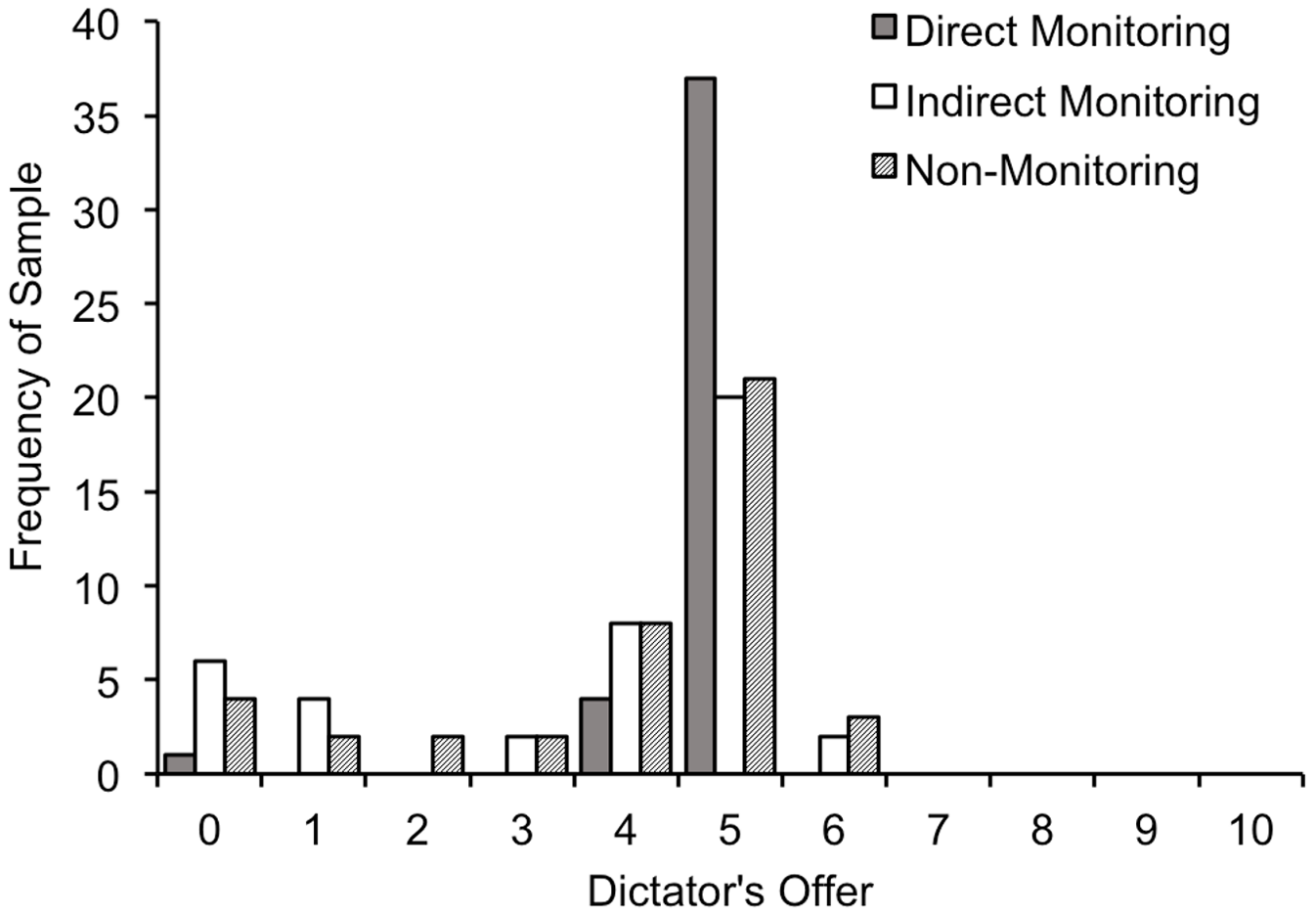
Distribution of dictator's offers by condition.

Fig. 1 shows the mean levels of allocation for each condition, and Fig. 2 shows the distributions of allocation for each condition. A one-way ANOVA revealed that the mean allocation levels for each condition were significantly different (*F_2, 82_* = 10.15, *p* < .001, *η_p_*^2^ = .198). A post-hoc *t*-test with Bonferroni correction revealed that there was no significant difference between the indirect monitoring and the non-monitoring conditions (*t*(41) = 1.59, *p* = .356, *d* = 0.17). However, the mean level of allocation in the direct monitoring condition was significantly higher than that of the non-monitoring condition (*t*(41) = 3.01, *p* = .013, *d* = 0.59).

While the mean pass rate in the first-order false belief task was 76% (32 out of 42), the mean pass rate in the second-order false belief task was only 12% (5 out of 41). One child did not engage in the second-order false belief task due to physical difficulties (Table S1). Furthermore, the performance of the second-order false belief task was not associated with the amount of allocation for any of the three conditions.

## Discussion

The purpose of this study is to examine the effect of direct and indirect monitoring on generosity among five-year-old preschoolers and to reveal the primary motivation for this generosity. While the five-year-old children in the direct monitoring condition provided more resources to their classmates than did the children in the non-monitoring condition, mean allocation levels between the indirect and the non-monitoring conditions were not significantly different. Furthermore, only 12% (5 out of 41) of the five-year-old children passed the second-order false belief task. These results indicate that five-year-old children are more concerned with the direct response they receive from others than they are about their own reputations.

According to previous developmental studies, five-year-old children will make fair allocations in order to avoid direct punishment from an opponent when playing a face-to-face economic game^20^,^21^. The children in this study might have thought that the adult experimenter would scold them for choosing in a selfish manner or that the experimenter would praise them for choosing in a kind manner; therefore, their mean resource allocation levels were higher in the direct monitoring condition than in the non-monitoring condition. Our results support the hypothesis that the primary motivation for five-year-old children's generosity is a concern for the direct responses they will receive from others, as opposed to concern for their own reputations.

While a number of studies have shown that the prominent display of images of eyes promote generosity in adult participants^4^,^5^,^6^,^8^ and enhance their motivation for building a good reputation^7^, our study did not show this result. Why was the effect of indirect monitoring on generosity not observed concerning preschoolers' generosity? As mentioned in the introduction, higher-order ToM is considered to be key in instigating the indirect monitoring effect. Since previous research found that this capability is acquired between the ages of eight and nine^17^, these older children would naturally be more concerned about their own reputations and would understand more about what others think about their actions. Perhaps the mechanism responsible for increased sensitivity to eye cues that is seen in the generosity of adults, but not in very young children, develops around the ages of eight or nine years. However, as five-year-old children were not concerned with their own reputations, they were only concerned with the direct responses they received from others, whether punishments or rewards. This suggests that children's generosity develops in two stages, as described above.

The results of this study are promising; however, there are certain problems with the methodology and some issues that need to be further addressed. As some recent studies reported no eye-image effect in adult participants' generosity^22^,^23^,^24^,^25^,^26^, it is necessary to carefully confirm the indirect monitoring effect on children's generosity. Therefore, future research should conduct this same experiment with older children (over nine years old) who are able to pass the second-order false belief task in order to confirm whether this indirect monitoring effect on generosity is observed among older children.

## Methods

### Participants

A total of 42 children (25 girls and 17 boys), all third graders in one private Japanese kindergarten, participated in this study, and the relevant data was collected for each of these children. The number of participants was determined by the kindergarten's enrolment. All participants were in the same class and grade, with a mean age of 5.3 (*SD* = 0.5). The ethical committee at Tamagawa University approved this study, and consent was obtained from all the students' parents and from their kindergarten teacher. The methods were carried out in accordance with the approved guidelines.

### Dictator Game

All children played the role of a dictator in a one-shot DG and were asked to divide 10 chocolates between themselves and an anonymous recipient. We used three within-subjects conditions: direct monitoring, indirect monitoring, and non-monitoring. All of the children played the DG in each of the three conditions on different days. The condition presentation order was randomized.

**Figure 3:**
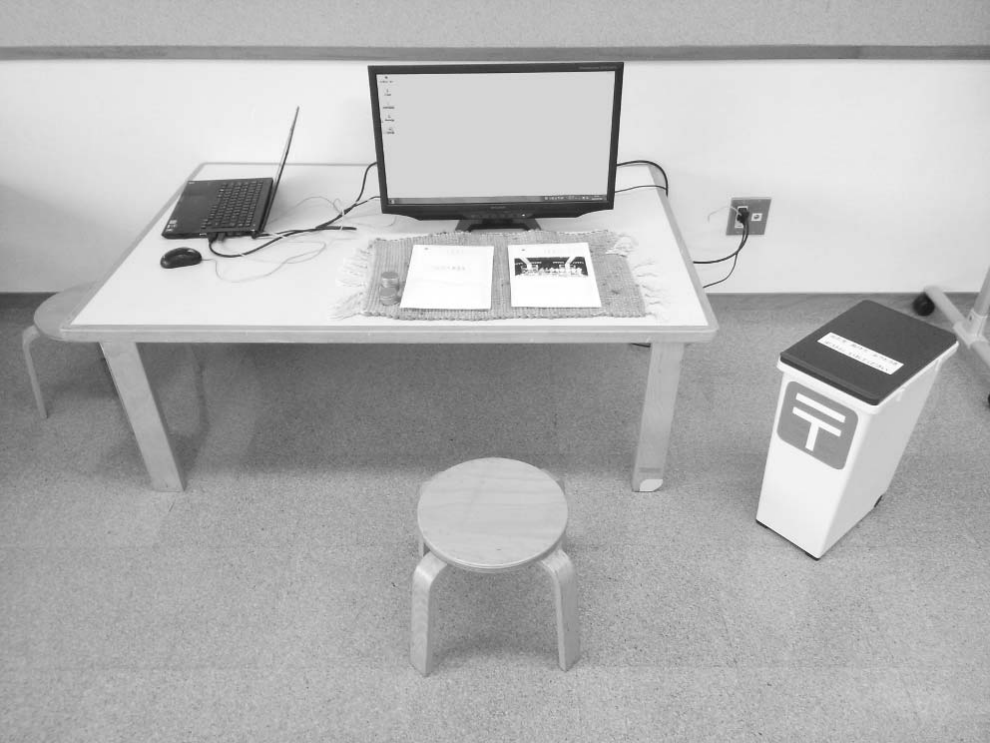
A photo of the experimental environment.

In the direct monitoring condition, ten chocolates, two trays, and a photo of all the classmates together were placed on the table (Fig. 3). The children were told that one tray belonged to them but the other belonged to their classmate. One classmate in the photo was randomly selected as the DG's recipient at the end of the experiment. After they received a full explanation of what they were expected to do in the task, the children completed the DG while a male experimenter watched them. The DG ended when the participant placed the sweets on the two trays.

In the indirect monitoring condition, two envelopes were placed on the table instead of two trays. In this condition, the photo of the classmates and the participant's name were attached to one of the two envelopes. An envelope was not used in the direct monitoring condition in order to enhance the feeling of being monitored and to prevent the children from cheating. A 23-inch computer monitor was placed in front of the children, and an image (a drawing of eyes resembling the makeup of a traditional Japanese Kabuki actor, which was the same image used by Mifune, et al.^6^UGOKU BOHAN NO ME, Tokyo Prefecture) was displayed on the monitor. After explaining the rules of the task and confirming that children saw the image of either the eyes or the flowers, the experimenter left the room, and the children completed the DG alone. The children put some sweets into the envelopes and sealed them. Then, they put two envelopes into the collection box and left the room. The procedure for the DG in the non-monitoring condition was the same as in the indirect monitoring condition, but a picture of flowers was used as the display stimulus instead of the pair of eyes. After the experiment, the children received the sweets according to their decisions.

### False Belief Task

To test for acquisition of theory of mind, we used first- and second-order false belief tasks. Children performed these tasks on a personal computer. We used an animated version of a false belief task (animated version of the Theory of Mind Task, DIK, Inc., http://www.kokoro-cd.com/). They watched a short movie clip (about one minute long) and answered questions concerning the first- and second-order false beliefs of the film's characters. We used the Sally-Anne Task^27^ as a first-order false belief task and the ice cream task^17^ as a second-order false belief task.

## Supplementary Material

Supplementary Information Dataset 1
