# Supplementary material for: The Effect of Direct and Indirect Monitoring on Generosity Among Preschoolers
Source: Sci Rep. 2015 Mar 12;5:9025. doi: 10.1038/srep09025 (PMC4356952; doi:10.1038/srep09025)
Supplement: Supplementary Information — Dataset 1 [file srep09025-s1.docx]

**Supplementary Information**

**The Effect of Direct and Indirect Monitoring on Generosity Among Preschoolers**

Takayuki Fujii, Haruto Takagishi, Michiko Koizumi, Hiroyuki Okada

Supplementary Table 1. Participant’s Behavioural Data

| Sex | Age | Direct  Monitoring | Indirect  Monitoring | Non  Monitoring | First order FBT  (Passed = 1, Failed = 0) | Second order FBT (Passed = 1, Failed = 0) |
| --- | --- | --- | --- | --- | --- | --- |
| F | 5 | 5 | 4 | 1 | 1 | . |
| F | 5 | 4 | 2 | 0 | 1 | 0 |
| F | 6 | 5 | 4 | 5 | 1 | 0 |
| M | 5 | 0 | 1 | 1 | 1 | 0 |
| F | 6 | 5 | 5 | 5 | 1 | 0 |
| M | 5 | 5 | 6 | 5 | 0 | 0 |
| M | 5 | 5 | 4 | 4 | 1 | 0 |
| F | 5 | 5 | 5 | 5 | 1 | 0 |
| F | 5 | 5 | 5 | 5 | 1 | 1 |
| F | 6 | 5 | 4 | 5 | 1 | 0 |
| F | 5 | 5 | 2 | 4 | 1 | 1 |
| F | 6 | 5 | 5 | 5 | 1 | 0 |
| F | 5 | 5 | 5 | 3 | 1 | 0 |
| F | 5 | 4 | 4 | 3 | 1 | 0 |
| M | 5 | 5 | 0 | 0 | 1 | 1 |
| F | 5 | 5 | 5 | 6 | 1 | 0 |
| M | 5 | 5 | 5 | 5 | 0 | 0 |
| F | 5 | 5 | 5 | 4 | 1 | 0 |
| M | 6 | 5 | 5 | 5 | 1 | 0 |
| F | 5 | 5 | 5 | 5 | 1 | 0 |
| M | 5 | 5 | 5 | 0 | 1 | 0 |
| F | 5 | 5 | 5 | 5 | 0 | 0 |
| M | 6 | 5 | 3 | 1 | 1 | 1 |
| M | 6 | 5 | 1 | 1 | 1 | 0 |
| M | 5 | 5 | 4 | 5 | 1 | 0 |
| M | 5 | 5 | 0 | 0 | 1 | 0 |
| F | 5 | 5 | 0 | 0 | 1 | 0 |
| M | 5 | 5 | 5 | 5 | 1 | 0 |
| M | 6 | 5 | 5 | 4 | 0 | 0 |
| F | 5 | 5 | 6 | 5 | 1 | 0 |
| F | 6 | 5 | 5 | 5 | 0 | 0 |
| M | 5 | 4 | 5 | 4 | 0 | 0 |
| M | 6 | 5 | 0 | 0 | 0 | 0 |
| F | 6 | 5 | 5 | 5 | 1 | 0 |
| F | 5 | 5 | 5 | 5 | 0 | 0 |
| F | 6 | 5 | 4 | 4 | 1 | 0 |
| F | 5 | 5 | 5 | 5 | 1 | 0 |
| F | 5 | 5 | 4 | 6 | 1 | 1 |
| M | 5 | 4 | 6 | 4 | 0 | 0 |
| M | 5 | 5 | 5 | 5 | 1 | 0 |
| F | 5 | 5 | 5 | 5 | 1 | 0 |
| F | 5 | 5 | 3 | 4 | 0 | 0 |

*FBT = False Belief Task
